# The polymorphism *G894 T* of endothelial nitric oxide synthase (*eNOS)* gene is associated with susceptibility to essential hypertension (EH) in Morocco

**DOI:** 10.1186/s12881-018-0638-1

**Published:** 2018-07-27

**Authors:** Sanaa Nassereddine, Hind Hassani Idrissi, Rachida Habbal, Rhizlane Abouelfath, Farah Korch, Majda Haraka, Adnane Karkar, Sellama Nadifi

**Affiliations:** 1 Laboratory of Genetics and Molecular Pathology, Medical School, University Hassan II, Casablanca, Morocco; 2 Department of Cardiology, University Hospital Center Ibn Rochd, Casablanca, Morocco

**Keywords:** Essential hypertension (EH), Susceptibility, *G894 T eNOS* polymorphism, Moroccan population

## Abstract

**Background:**

Hypertension is a multifactorial disease involving both environmental and genetic Factros. *G894 T eNOS* polymorphism has been suggested to be responsible for reduced NO synthesis, and EH development. The objective of our case-control study is to evaluate the potential association of *G894 T eNOS* polymorphism with Essential Hypertension (EH) susceptibility, among a sample of Moroccan patients.

**Methods:**

One hundred forty five hypertensive patients were recruited from the department of Cardiology, University Hospital Center Ibn Rochd, Casablanca, Morocco, and compared to 184 apparently healthy subjects. DNA samples were genotype by PCR-RFLP method using *MboI* restriction enzyme.

**Results:**

Our results showed a positive correlation between G894 T eNOS distribution and Alcohol and Obesity rik factors (*P* = 0.009 and 0.02 respectively). Patients with elevated Cardio Vascular Risk (CVR) carried out the higher frequency of homozygous mutant genotype TT (62.2%) and T mutant allele (77.8%), compared to median and low CVR groups. *G894 T eNOS* distribution was significantly associated to a high risk of EH occurrence under the GT and TT genotypes (OR [95% CI] = 20.2 [7.7–52.4], *P* <  0.0001; OR [95% CI] = 332.5 [98.2–1125.4], *P* <  0.0001 respectively), and the 3 genotypic transmission models (**Dominant**: OR [95% CI] = 43.2 [17.9–104.09], *P* <  0.0001; **Recessive**: OR [95% CI] = 47.7 [18.6–122.3]; *P* <  0.0001; **Additive:** OR [95% CI] = 14.02 [9.6–20.45], *P* <  0.0001).

**Conclusion:**

Our study suggests a strong association of *G894 T eNOS* polymorphism with susceptibility to EH in Morocco. Studies trying to identify contributing genes may be very useful and allow recognizing the vulnerable individuals and classifying patients in subgroups with definite genetic and pathogenic mechanisms to achieve better prevention and therapeutics.

## Background

Hypertension is a multifactorial disease involving both environmental and genetic components. Alterations in endothelial-derived Nitric Oxide (NO) production have been associated with numerous diseases [1, 2], and, in humans, can be genetically determined by the presence of different polymorphisms in the *eNOS* gene.

Synthesized by endothelial cells, NO contributes to the vasodilatation process and regulation of Blood Pressure (BP) [1]. Its production in vascular endothelium cells is controlled by endothelial Nitric Oxide Synthase (eNOS) gene, from L-arginine oxidation [3].

**Fig. 1:**
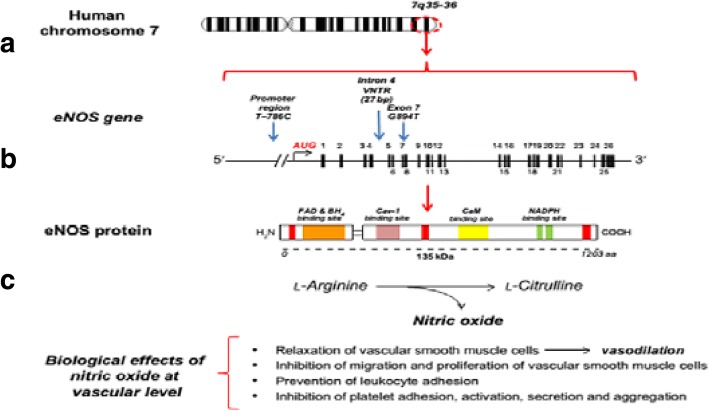
Scheme of the gene encoding endothelial nitric oxide synthase gene (eNOS) (**a** and **b**) [18]. Scheme of the gene encoding endothelial nitric oxide synthase (eNOS or NOS3). The human NOS3 gene (located at 7q35-36) contains 26 exons that span 21 kb. Exons are described by number. AUG: the transcription start site. Three specific polymorphisms in the NOS3 gene are marked by arrows. **c** Scheme of the eNOS protein

The gene encoding eNOS is located on chromosome 7q35–36, and composed of 25 introns and 26 exons that encode a 135 KDa protein, containing 1203 amino acids (Fig. 1) [4, 5]. *eNOS* gene is highly polymorphic. The G894 T (Glu298Asp) variant, located in exon 7, is the most described and has been suggested to be responsible for reduced NO synthesis, and EH development [6]. This variant alters the primary structure of the protein and has the potential to alter one or more functional properties of the enzyme directly. Two different studies have shown the eNOS protein containing Asp at position 298 to be subject to selective proteolytic cleavage in endothelial cells and vascular tissues [7, 8]. If this observation is correct, the cleaved fragments would be expected to lack NO synthase activity. However, two other reports suggest that this observation might be an artifact [9, 10]. The Glu298Asp SNP affects also eNOS localization to caveolar membrane [11].

As there is lack of data concerning the potential association of G894 T eNOS polymorphism with EH susceptibility in North African populations and especially in the Moroccan one, we have preceded to the evaluation of its effect on EH predisposition in a sample of Moroccan patients compared to healthy subjects.

## Methods

### Study population

Blood samples were collected from 145 hypertensive patients, recruited from the department of Cardiology, University Hospital Center Ibn Rochd, Casablanca, Morocco, and compared to 184 apparently healthy subjects. Patients with a mean systolic blood pressure (SBP) ≥140 mmHg, mean diastolic blood pressure (DBP) ≥90 mmHg or taking antihypertensive therapy were declared hypertensive and were recruited. However, those presenting symptoms or signs suggesting secondary origins of hypertension were excluded from the study. For healthy subjects, they did not show any abnormalities concerning the physical status, blood pressure and family history of hypertension. Patients’ Clinical data were collected and an informed consent, approved by the Ethical Committee of the University of Hassan II, School of Medicine, Casablanca, was signed by each patient and control before entering the study.

### DNA extraction

Venous blood from all participants in this study was collected in EDTA tubes. Samples were stored at − 20 °C until extraction of DNA. Genomic DNA was extracted from blood leukocytes using the standard method of salting out [12].

### Genotype determination

**Fig. 2:**
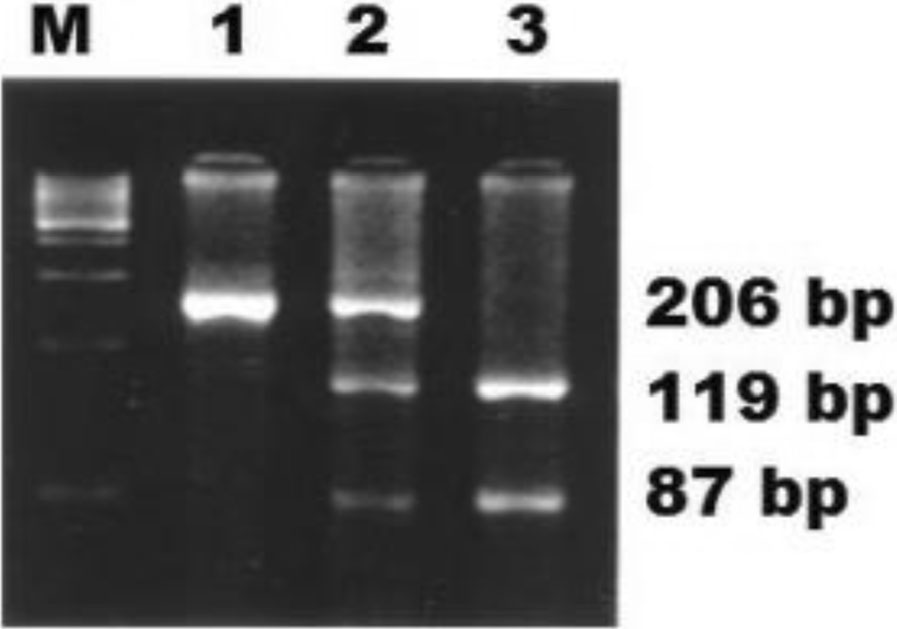
PCR-RFLP analysis on agarose gel of G894T eNOS polymorphism. M: size marker; Lane 1: GG; Lane 2: GT; Lane 3: TT

We used PCR-RFLP to genotype samples for +894G/T eNOS polymorphism as previously described by [13]. We have proceeded to an amplification of 50–100 ng of extracted DNA, followed by digestion using *MboI* restriction enzyme, which gave rise to three profiles: homozygous wild type GG (one fragment of 206 bp), heterozygous GT (three fragments of 206, 119 and 87 bp), and homozygous mutated TT (two fragments of 119 and 87 bp) (Fig. 2). The digested products were separated on 3% agarose gel electrophoresis stained with Ethidium Bromide (BET), and visualized with UV rayons.

### Statistical analysis

Statistical analysis was performed using SPSS 21.0 software. Chi square test (χ2) and a *P* value < 0.05 were used to determine statistical significance of association/non-association between genotypes and classical risk factors. Hardy–Weinberg Equilibrium test (HWE) was performed in both cases and controls groups for the analyzed polymorphism. Odd ratio (OR) was calculated to estimate the association between genotypes and EH risk, using a Confidence Interval (CI) of 95%. Significance was approved at *P*-value less than 0.05.

## Results

Genotypic distribution of the *G894 T eNOS* polymorphism was in Hardy-Weinberg Equilibrium (HWE) among both cases and controls (Table 1). The average age of our patients was 61.15 ± 10.23 Vs 54.15 ± 2 for healthy subjects. There was a predominance of female in the cases group compared to male (80% Vs 20% respectively).

**Table 1:** *G894 T eNOS* distribution Vs Clinical parameters

	eNOS			*P* value (< 0.05)
	GG %	GT %	TT %	
Age	58.6 ± 9.34	61.77 ± 10.1	60.87 ± 10.25	0.07
Gender				0.4
♀	2.6	39.7	57.7	
♂	6.9	34.5	58.6	
Ethnicity				0.5
White	4.5	41	54.5	
Metis	3.4	34.8	61.8	
Black	0	58.3	41.7	
Familial History of EH				0.5
yes	4.5	39.8	55.7	
no	1.8	36.8	61.4	
Familial History of CVD				0.6
yes	5.9	38.2	55.9	
no	2.7	38.7	58.6	
Familial History of Cerebrovascular disease				0.9
yes	2.6	36.9	60.5	
no	3.7	39.3	57	
Familial History of Diabetes				0.3
yes	3.8	43.6	52.6	
no	3	32.8	64.2	
Familial History of Renal disease				0.8
yes	0	40	60	
no	3.7	38.5	57.8	
Familial History of Dyslipidemia				0.3
yes	4.7	30.2	65.1	
no	2.9	42.2	54.9	
Smoking				0.2
yes	7.7	53.8	38.5	
no	3	37.1	59.9	
Alcohol				0.009*
yes	25	75	0	
no	2.8	37.6	59.6	
Physical Activity				0.05
yes	7.1	35.7	57.1	
no	0	41.3	58.7	
Obesity				0.02*
yes	6.5	27.4	66.1	
no	1.2	47	51.8	
Dyslipidemia				0.9
yes	3.4	37.9	58.6	
no	3.4	39.1	57.5	
Diabetes				0.9
yes	3.8	37.7	58.5	
no	3.3	39.1	57.6	
Menopausal status				0.2
yes	3	32.8	64.2	
no	2	48	50	
Personal History of CVD				0.07
yes	9.7	29	61.3	
no	1.9	42.9	55.2	
Nephropathy				0.8
yes	0	33.3	66.7	
no	3.6	38.8	57.6	
Nbr of antihypertensive molecules				0.7
1	3.1	37.5	59.4	
2	5.1	42.3	52.6	
3	0	36	64	
4	0	22.2	77.8	
TA				0.7
controled	4.3	37.2	58.5	
non-controled	2	41.2	56.8	
Neurological deficit				0.7
yes	0	33.3	66.7	
no	3.7	39	57.3	
Electrical HVG				0.7
yes	0	38.5	61.5	
no	3.8	38.6	57.6	
Repolarisation troubles				0.5
yes	5.8	47.1	47.1	
no	3.1	37.5	59.4	
Creatinine	7.9 ± 4.14	11.38 ± 6.21		0.2
TG				0.5
≤ 1	4.5	41	54.5	
> 1	2.4	31.3	66.3	
CT				0.3
≤ 1	0	0	3	
> 1	2.8	36.7	60.6	
HDL				0.8
≤ 0.5	2	36	62.8	
> 0.5	3.8	32.1	64.2	
LDL				0.9
≤ 0.5	0	0	100	
> 0.5	3	31	66	
Glucose	1.02 ± 0.48	1.18 ± 0.56	1.32 ± 0.6	0.9
Na+	137 ± 28	139.6 ± 34.9	135.3 ± 20.6	0.3
K+	4.1 ± 1.8	4.06 ± 1.9	4.22 ± 1.8	0.3

*Statistically significant; *Nbr* number, *HVG* left ventricular hypertrophy, *GG* Homozygous wild type, *GT* Heterozygous, *TT* Homozygous mutant

Table 1 shows the correlation between clinical parameters of our patients and their *G894 T eNOS* genotypes distribution. There was a statistically significant association only with Alcohol (*P* = 0.009) and Obesity (*P* = 0.02); a tendency to a significant association was found with age (*P* = 0.07), Physical activity (*P* = 0.05) and personal cardiovascular history (*P* = 0.07).

Correlation of cardiovascular risk (CVR) status to *G894 T eNOS* allelic and genotypic distributions, shows no statistically significant association (*P* = 0.5), even patients with elevated CVR carried out the higher frequency of homozygous mutant genotype TT (62.2%) and T mutant allele (77.8%), compared to median and low CVR groups (Table 2).

**Table 2:** *G894 T eNOS* distribution Vs Cardio Vascular Risk

	eNOS					*P* value (< 0.05)
	GG %	GT %	TT %	G allele %	T allele %	
CVR Status						0.5
Low	2.3	43.2	54.5	23.9	76.1	
Median	1.8	41.1	57.1	22.2	77.6	
High	6.7	31.1	62.2	22.2	77.8	

*CVR* cardio vascular risk, *GG* Homozygous wild type, *GT* Heterozygous, *TT* Homozygous mutant

Table 3 reports the allelic and genotypic distribution of *G894 T eNOS* polymorphism, among cases and controls. Our results showed that 77.93% of patients were carrying the mutant allele T, 59.27% of them having the homozygous mutant profile, and 37.24% that were heterozygous. The majority of healthy subjects (79.89%) carried out the wild type allele G, with 63.04% of them having the homozygous wild type genotype GG. *G894 T eNOS* polymorphism was significantly associated to a high risk of HTA occurrence under the GT and TT genotypes (OR [95% CI] = 20.2 [7.7–52.4], *P* <  0.0001; OR [95% CI] = 332.5 [98.2–1125.4], *P* < 0.0001 respectively), and also the 3 genotypic transmission models (**Dominant**: OR [95% CI] = 43.2 [17.9–104.09], *P* < 0.0001; **Recessive**: OR [95% CI] = 47.7 [18.6–122.3]; *P* < 0.0001; **Additive:** OR [95% CI] = 14.02 [9.6–20.45], *P* < 0.0001).

**Table 3:** Allelic and genotypic distribution of *G894 T eNOS* polymorphism among cases and controls

		*N* = 145	*N* = 184		
		Cases N(%)	Controls N(%)	OR [95% CI]	*P* value (< 0.05)
Genotypes	GG	5 (3.49%)	116 (63,04)	1	
	GT	54 (37.24%)	62 (33,69)	20.2 [7.7–52.4]	< 0.0001*
	TT	86 (59.27%)	6 (3,27)	332.5 [98.2–1125.4]	< 0.0001*
Dominant model	GG + GT	59 (40.73%)	178 (96,73)	1	
	TT	86 (59.27%)	6 (3,27)	43.2 [17.9–104.09]	< 0.0001*
Recessive model	GG	5 (3.49%)	116 (63,04)	1	
	GT + TT	140 (96.51%)	68 (36,96)	47.7 [18.6–122.3]	< 0.0001*
Additive model	G	64 (22.07%)	294 (79,89)	1	
	T	226 (77.93%)	74 (20,11)	14.02 [9.6–20.45]	< 0.0001*

*Statistically significant; *GG* Homozygous wild type, *GT* Heterozygous, *TT* Homozygous mutant

## Discussion

The human *eNOS* gene is highly polymorphic. Results about the association of *eNOS* Single Nucleotide Polymorphisms with Essential Hypertension development are often controversial and inconclusive [14–18]. Many investigators studied the relationship between *G894 T eNOS* polymorphism and hypertension. There is an apparent discrepancy among results in these association studies [19–25]. It was found to be associated with increased risk of hypertension in Caucasians [23, 24], but in another two studies it was not (in neither Caucasians nor Japaneses) [26, 27].

NO is known to be an endothelium-derived relaxing factor and a local blood flow regulator in healthy humans [23, 24, 28]; these facts led to the hypothesis that a reduction in the activity of the endothelial L-arginine–NO pathway could initiate or contribute to the development of Essential Hypertension by promoting and sustaining the observed increase in peripheral resistance [14, 29].

Some researchers demonstrated that inhibition of *eNOS* elevates Blood Pressure in healthy humans, and disruption of the *eNOS* gene leads to Hypertension in mice [15, 16]. Analyses of genetically engineered animals deficient in eNOS expression (eNOS^**−**^/^**−**^) provide support for the importance of eNOS-derived NO, especially in cardiovascular homeostasis. For instance, eNOS^**−**^/^**−**^ mice develop numerous vascular disease states, including systemic hypertension [15, 30]. Moreover, patients with Essential Hypertension have either diminished whole body NO production or increased inactivation leading to lower plasma levels [31–34]. These results strongly implicate genetic alterations in the *eNOS* gene in the pathogenesis of human EH [35].

To investigate the potential implication of *G894 T eNOS* polymorphism in EH, we performed a case-control study among a sample of Moroccan EH patients. Our results showed –first- that Alcohol and obesity were the clinical parameters significantly associated with *G894 T eNOS* distribution (*P* = 0.009 and 0.02 respectively); a tendency to a statistically significant association was also found with ‘Age’, Physical activity’ and ‘Personal history of cardiovascular disease’ (*P* = 0.07, 0.05 and 0.07 respevtively) (Table 1). As known, the pathogenesis of hypertension may be explained by the interaction of many factors such as sodium intake, overweight, alcohol, smoke and the genetic background of subjects [36, 37].

Correlation between cardiovascular risk (CVR) status and *G894 T eNOS* distribution showed no statistically significant association (*P* = 0.5); even patients having elevated CVR carried out the higher frequency of mutant homozygous genotype TT (62.2%) (Table 2).

We found a statistically very significant association of this variant of *eNOS* gene with increased risk of EH development in our study sample, under the GT and TT genotypes (OR [95% CI] = 20.2 [7.7–52.4], *P* < 0.0001; OR [95% CI] = 332.5 [98.2–1125.4], *P* < 0.0001 respectively), and also the 3 genotypic transmission models (**Dominant**: OR [95% CI] = 43.2 [17.9–104.09], *P* < 0.0001; **Recessive**: OR [95% CI] = 47.7 [18.6–122.3]; *P* < 0.0001; **Additive:** OR [95% CI] = 14.02 [9.6–20.45], *P* < 0.0001). However, studies results about the association of this variant with susceptibility to EH are controversial and divergent [14–17]. Many of these studies suggested that inhibition of *eNOS* pathway lead to BP elevation by low NO production [14, 29]. Study of Wenru Tang et al.*..* [35], analyzing the association of *G894 T eNOS* polymorphism with EH predisposition, among two Chinese populations (Hani and Yi), reported that –in Hani population- the *894 T* allele was associated with increased risk of pathology development, while, in Yi population, the association was found with the *894G* allele. Shoji M et al [21] also reported an association of the *894 T* allele with EH susceptibility and elevated BP among Japanese subjects. Several studies reported similar results [23, 28], when others found controversial results [19, 20, 22, 26, 27, 38].

This inconsistency in results can be explained by the fact that many of these studies didn’t take in consideration the rest of risk factors that may influence the susceptibility to EH, such as ethnicity (Table 4), age, gender, obesity… [39]. eNOS enzyme acts as a homodimer, functionally composed of two major domains: a C-terminal reductase domain and an N-terminal oxygenase domain. Besides the cofactors tetrahydrobiopterin, flavin adenine dinucleotide, flavin mononucleotide, and calmodulin, eNOS catalytic activity requires the presence of a heme domain [40]. Studies analyzing the structure of eNOS protein have demonstrated that the Glu298 variant of *eNOS* humain gene is located on the catalytic site of the heme part of the protein, in contact with the protein-protein interaction zone, sensitively affected by Glu298Asp substitution [41]. In his study, Tesauro et al. suggests that the *Glu298* and *Asp298* variants are differentially treated in cells: Asp298 is prone to natural cleavage by proteases that cleave the *eNOS* protein in the *Glu/Asp 298* substitution site [42]. These findings suggest that *Glu/Asp 298* substitution may contribute to the pathogenesis of EH and modulate the therapeutic response of some patients against anti-hypertensive agents [43], leading to different BP responses and cardiovascular outcomes [44, 45].

**Table 4:** Distribution of G894 T eNOS polymorphism among different populations

Population	GG %	GT %	TT %	Reference
Egypt	50	40	10	[46]
Germany	50.5	40	9.5	[47]
Turkish	49.3	41.3	9.3	[48]
English	47.8	42	10.2	[13]
Japan	84.4	17.4	0	[22]
Korea	97.6	19.5	0.9	[49]
Caucasians	50.3	39.5	8.2	[50]
African Americans	70.4	23.9	5.6	[51]
South African	78.6	19	2.4	[52]
Our study	63.06	33.69	3.27	[53]

*GG* Homozygous wild type, *GT* Heterozygous, *TT* Homozygous mutant

M: DNA size marker; Lane 1: GG; Lane 2: GT; Lane 3: TT

In the present study, some limitations have to be noted; first the small sample size of patients and controls that was analyzed (145 and 184 respectively); the lack of serum NO concentration measurement; It should also be noted that hypertension is a complex disease; other candidate genes may contribute to the susceptibility of hypertension, and thus must be analyzed. Further studies including larger sample sizes, and overcoming the current limitations might be very useful to better understand the genetic predisposition to hypertension in our population.

## Conclusion

Our study is the first in Morocco to evaluate the association of *G894 T eNOS* polymorphism with EH risk of occurrence. Our results suggest a strong association of this variant of *eNOS* gene with EH susceptibility in our study sample. Genetic factors contribute to 30–50% of BP variability in human EH. Studies trying to identify contributing genes may provide useful information and allow recognizing the vulnerable individuals and classifying patients in subgroups with definite genetic and pathogenic mechanisms to achieve better prevention and therapeutics. In this context, further analyses still be needed, in order to investigate eNOS adjacent markers in a wider context; future studies should also focus on gene–gene and gene–environment interactions, as well as haplotype patterns.

## Abbreviations

BP: Blood Pressure
CI: Confidence Interval
EH: Essential Hypertension
eNOS: endothelial oxide nitric synthase
G894 T eNOS: Substitution G/T on 894 position of eNOS gene sequence
OR: Odds Ratio
PCR: Polymerase Chaine Reaction
RFLP: Restriction Fragment Length Polymorphism
